# Synergy Between Proline-Rich Antimicrobial Peptides and Small Molecule Antibiotics Against Selected Gram-Negative Pathogens *in vitro* and *in vivo*

**DOI:** 10.3389/fchem.2018.00309

**Published:** 2018-08-14

**Authors:** Laszlo Otvos Jr., Eszter Ostorhazi, Dora Szabo, Steven D. Zumbrun, Lynda L. Miller, Stephanie A. Halasohoris, Puvi D. Desai, Sharon M. Int Veldt, Carl N. Kraus

**Affiliations:** ^1^OLPE, LLC, Audubon, PA, United States; ^2^Institute of Medical Microbiology, Semmelweis University, Budapest, Hungary; ^3^Arrevus, Inc., Raleigh, NC, United States; ^4^Bacteriology Division, United States Army Medical Research Institute of Infectious Diseases Fort Detrick, MD, United States

**Keywords:** carbapenems, colistin, enzyme inhibition, melioidosis, resistant bacteria, synergy, systemic infection, ARV-1502

## Abstract

As monotherapy, modified proline-rich antimicrobial peptides (PrAMPs) protect animals from experimental bacteremia in a dose-dependent manner. We evaluated the *in vitro* synergy of a modified PrAMP, A3-APO, a dimer, previously shown to inhibit the 70 kDa bacterial heat shock protein DnaK, with imipenem or colistin against two antibiotic-resistant pathogens; a carbapenemase-expressing *Klebsiella pneumoniae* strain K97/09 and *Acinetobacter baumannii* (ATCC BAA-1605). Combining antimicrobials resulted in synergy for PrAMP/colistin combination against both *K. pneumoniae* and *A. baumannii* (ΣFIC = 0.08 both) and additive activity for the A3-APO/imipenem combination against *K. pneumoniae* (ΣFIC = 0.53). Chex1-Arg20, (designated as ARV-1502 in preclinical development), the single chain PrAMP monomer of A3-APO, showed synergy with meropenem against a carbapenem-resistant uropathogenic *Escherichia coli* strain (ΣFIC = 0.38). In a murine bacteremia model using K97/09, A3-APO at 1 mg/kg demonstrated improved survival when co-administered with standard (10 mg/kg) or subtherapeutic (1 mg/kg) doses of colistin at 36 h (*p* < 0.05). Surprisingly, the survival benefit of A3-APO was augmented when the A3-APO dose was decreased by 50% to 0.5 mg/kg (*p* < 0.02) in conjunction with a subtherapeutic colistin dose (1 mg/kg). ARV-1502, as monotherapy demonstrated prolonged (>24 h) activity in a mouse *Escherichia coli* infection assay. Co-treatment with ARV-1502 and subtherapeutic doses of ceftazidime (150 mg/kg) was studied in a mouse model of melioidosis. ARV-1502 provided a 50% improvement in long-term (62 days) survival, but only at the lowest of 3 administered doses; survival advantage was demonstrated at 2.5 mg/kg but not at 5 or 10 mg/kg. The mortality benefit of combination therapies was not routinely accompanied by a parallel decline in blood or tissue bacterial counts in surviving animals, suggesting that the anti-infective activity of the host defense peptides (HDP) is broader than simply bacterial eradication. In fact, the hormetic effect observed in either animal models suggest that low dose HDP treatment may change the dominant mode of action in experimental bacteremia.

## Introduction

Modified proline-rich antimicrobial peptides (PrAMPs) have repeatedly been shown to protect mice from Gram-negative bacteremia (Knappe et al., 2012; Schmidt et al., 2016). The dimeric PrAMP, A3-APO, exhibits such protective effects against *Escherichia coli* and *Acinetobacter baumannii* in a dose-dependent manner (Szabo et al., 2010; Ostorhazi et al., 2011a). Nevertheless, the sub-optimal therapeutic index (TI) of host-defense peptides (HDP) (Bush et al., 2004) when administered intravenously (iv) requires that alternative modes of administration be evaluated. In a detailed study of the *in vivo* toxicity parameters of a designer HDP called DP7, 20 mg/kg iv administration was found to result in 33% of the mice surviving due liver hyperemia (Wu et al., 2014). DP7 administered subcutaneously (sc) also leads to hemorrhaging at the injection site, while no health problems are observed after intraperitoneal (ip) or intramuscular (im) administration. Indeed, im administration not only improves the toxicity profile for both A3-APO and its single chain metabolite, Chex1-Arg20 (commercially being developed as ARV-1502), but the peptides also demonstrate enhanced potency with this route of administration (Ostorhazi et al., 2011a, 2013). After im administration, the TI of the A3-APO in mice is 25 (Ostorhazi et al., 2010). In a preclinical study when administered im, the monomer exhibits no observed adverse effect limits of 30 mg/kg in rats and 4 mg/kg in dogs, translating to TI values of 30 and 12 in allometric therapy doses in the given species, respectively (unpublished data).

The lack of positive predictive value between *in vitro* microbiological activity and *in vivo* efficacy of the PrAMP suggests that the predominant mode of action is not direct bacterial killing; rather, the mechanism of action is likely related to the activation of host defense mechanisms (Ostorhazi et al., 2011b). This hypothesis is supported from many prior studies of HDP and relevant infection models (Nijnik et al., 2010; Hilchie et al., 2013; Otvos, 2016).

HDP exhibit a plethora of activities on hosts and invading bacterial cells (Brandenburg et al., 2012). A well-established option for reducing the therapeutic dose of PrAMPs, as well as small molecule antibiotics alike, is combination therapy (Cassone and Otvos, 2010). In most of these models, bacteria are killed by the legacy antibiotic and the PrAMP helps the legacy antibiotic in a variety of ways, ranging from disrupting bacterial membranes to inactivating bacterial housekeeping proteins. The potential clinical utility of PrAMP/antibiotic co-treatment has been described by several investigators evaluating bacteremia animal models (Cirioni et al., 2008; Hu et al., 2015). Peptide A3-APO inhibits the bacterial heat shock protein DnaK (Kragol et al., 2001). This inhibitory activity has the downstream effect, through limiting appropriate bacterial protein folding, of inactivating enzymes responsible for bacterial antibiotic resistance as well as bacterial toxin production (Otvos et al., 2006, 2014). Simultaneous incubation of *Escherichia coli, Klebsiella pneumonia*, and *Salmonella typhimurium* with A3-APO and amoxicillin, trimethoprim, chloramphenicol, or sulfonamides overcomes resistance provided by β-lactamases, dihydrofolate reductase, chloramphenicol acetyltransferase, and tetrahydropteroic synthetase respectively (Cassone et al., 2008). Indirect evidence from preliminary studies suggest that Chex1-Arg20, the *in vivo* monomeric metabolite of A3-APO (commercially developed as ARV-1502), binds bacterial DnaK better than its parent dimeric form (Cassone et al., 2008). The enhanced DnaK binding seems to predict enhanced *in vivo* activity since ARV-1502 is superior to A3-APO in both systemic and local animal infection models (Ostorhazi et al., 2011a, 2013).

Clinically, mitigation of necessary, but nephrotoxic antimicrobials such as colistin, through combination with ARV-1502 and subsequent antibiotic dose-reduction, has the potential to widen such compounds' therapeutic index. This is certainly a salient issue for infections that require prolonged treatment regimens such as in meliodosis. In the current report we investigated how either the monomeric modified PrAMP, Chex1-Arg20 (ARV-1502), or its dimeric form, A3-APO, enhances the potency of carbapenems, colistin, or ceftazidime against Gram-negative bacteria, either using *in vitro* assays or animal infection models. While mPrAMP co-treatment improved survival in all cases, a reduction in bacterial burden among surviving animals was not typical. Surprisingly, lower PrAMP doses resulted in enhanced survival compared to higher PrAMP doses. We postulate that these findings may be consistent with dose-dependent variable modes of action, reflecting a clinical candidate demonstrating hormesis, expanding the therapeutic index.

## Materials and methods

### *In vitro* studies

#### Bacterial strains

The *K. pneumoniae* strain used in this study originated from a human wound infection at Miskolc Healthcare Center/Semmelweis University Hospital and is designated as K97/09 (Toth et al., 2010). K97/09 is a carbapenemase-expressing strain (*bla*_KPC−2_) that is extensively drug-resistant, including ceftazidime, ceftriaxone, imipenem, meropenem, ciprofloxacin, gentamicin, and colistin. The *A. baumannii* strain (ATCC BAA-1605) used in this study originated from the tracheal aspirate of a Canadian soldier with ventilator-associated pneumonia. The strain is resistant to ceftazidime, gentamicin, piperacillin, aztreonam, cefepime, ciprofloxacin, imipenem, and meropenem (Tien et al., 2007). The *E. coli* UNT167-1 is a carbapenem resistant strain, isolated from a chronic urinary tract infection case at the University of Texas (Zhanel et al., 2018). The *B. pseudomallei* strain used in this study, 1026b, was originally isolated in 1993 from a 29 year old diabetic rice farmer in Thailand with melioidosis.

#### Antimicrobials

##### Peptides

A3-APO [(H-Chex-Arg-Pro-Asp-Lys-Pro-Arg-Pro-Tyr-Leu-
Pro-Arg-Pro-Arg-Pro-Pro-Arg-Pro-Val-Arg)_2_-Dab],
Chex1-Arg20 (H-Chex-Arg-Pro-Asp-Lys-Pro-Arg-Pro-
Tyr-Leu-Pro-Arg-Pro-Arg-Pro-Pro-Arg-Pro-Val-Arg-NH_2_)
and
Gly11 [(H-Chex-Arg-Pro-Asp-Lys-Pro-Arg-Pro-Tyr-Leu-Gly-
Arg-Pro-Arg-Pro-Pro-Arg-Pro-Val-Arg)_2_-Dab-NH_2_]

were gifts from Dr. Daniel Knappe, Leipzig University, Germany. The negative control leptin receptor antagonist Allo-aca (Otvos et al., 2011) used to confirm that an unrelated peptide has no activity, was a gift from Senn Chemicals, Dielsdorf, Switzerland. Gly11, which has the same amino acid sequence as A3-APO except for a change in one residue, fails to bind DnaK and was used to validate DnaK binding as critical to the mechanism of action of A3-APO (Cassone et al., 2008).

The colistin sulfate preparation (15,000 IU/mg) was from Sigma-Aldrich Kft (Budapest, Hungary) and imipenem was from MSD Budapest, Hungary Merck (tienamycin-formamidine-monohydrate sodium cilistatin marketed as Tienam).

#### *In vitro* activity and synergy

Minimal inhibitory concentration (MIC) assays were performed using sterile 96-well polypropylene plates in a final volume of 100 mL. Briefly, 50 μL of mid-logarithmic phase bacterial cultures were diluted to 5 × 10^5^ CFU/mL in Mueller-Hinton broth (MHB) and then added to 50 μL of the serially diluted antibiotic. The highest A3-APO and ARV-1502 concentration evaluated was 256 mg/L. Cultures were then incubated at 37°C for 16–20 h without shaking. MICs were identified as the lowest antimicrobial concentrations at which turbidity was not observed. Antimicrobial synergy was determined by evaluating the fractional inhibitory concentration (FIC) index and was characterized by a conventional checkerboard assay (Fernandez-Cuenca et al., 2003). Bacteria grown to mid-logarithmic phase in MHB were pre-incubated with serially diluted concentrations of peptides A3-APO or ARV-1502 and the antimicrobial controls, imipenem, colistin, or meropenem.

The sum of the FICs (ΣFIC) was calculated with the equation ΣFIC = FIC_A_ + FIC_B_ = (C_A_/MIC_A_) + (C_B_/MIC_B_), where MIC_A_ and MIC_B_ are the MICs of antimicrobial A and B alone, respectively, and C_A_ and C_B_ are the concentrations of the drugs when combined, respectively. Synergy was defined as a ΣFICs ≤ 0.5 and additive activity was defined as a ΣFICs > 0.5 ≤ 1.0.

### *In vivo* studies

#### Animals. assays 1–2 and ARV-1502 monotherapy

NMRI (Naval Medical Research Institute) BR or CD-1 mice (Toxi-Coop Zrt, Budapest, Hungary) were housed in plastic type 2 cages, 3–5 mice per cage, on softwood granules as bedding. The room was kept between 21 and 25°C with 12 h light:12 h dark cycles. The animals had free access to tap water and pelleted rodent food. Upon completion of the experiments, surviving mice were euthanized by diethyl ether inhalation. Animals were maintained and handled in accordance with the recommendations of the Guidelines for the Care and Use of Laboratory Animals, and the protocols were approved by the Animal Care Committee of Semmelweis University. The planned 15 treatment groups of mice were divided roughly equally into two assays with 8 and 7 treatment groups and untreated controls in each assay for safe and humane handling of large numbers of mice.

#### Infection models

NMRI mice weighing ~20 g (4 weeks old) were infected by intraperitoneal (ip) injection of 4x10^8^ CFU/g *K. pneumoniae* K97/09. Mice were randomly allocated to 8 and 9 groups (5 mice per group). Dosing is summarized in Supplementary Table 1.

Bacteremia synergy Assay 1:
*Group 1*: phosphate buffered saline (PBS) subcutaneously (sc) 1 h after infection.
*Group 2*: imipenem 30 mg/kg sc at 2, 14, and 26 h after infection.
*Group 3*: A3-APO 1 mg/kg im 1, 13, and 25 h after infection, imipenem 30 mg/kg sc at 2, 14, and 26 h after infection.
*Group 4*: colistin 10 mg/kg sc at 2, 14, and 26 h after infection.
*Group 5*: A3-APO 1 mg/kg im 1, 13, and 25 h after infection, colistin 10 mg/kg sc at 2, 14, and 26 h after infection.
*Group 6*: A3-APO 0.5 mg/kg im 1, 13, and 25 h after infection, colistin 10 mg/kg sc at 2, 14, and 26 h after infection.
*Group 7*: A3-APO 1 mg/kg im 1, 13, and 25 h after infection, colistin 1 mg/kg sc at 2, 14, and 26 h after infection.
*Group 8*: A3-APO 0.5 mg/kg im 1, 13, and 25 h after infection, colistin 1 mg/kg sc at 2, 14, and 26 h after infection.
*Group 9*: colistin 10 mg/kg sc 2 h after infection, A3-APO 1 mg/kg im 5 h after infection.

Survival was recorded hourly 24–36 h after infection. Blood samples (10 μL) were taken from the tail vein to determine the bacterial burden at 6 and 30 h after infection from all surviving animals. Groups with 2 or more animal having blood bacterial counts below the level of detection (1 × 10^3^ CFU/mL) at 6 h post-infection were excluded from analysis due to presumption of low inoculum or rapid host clearance. The blood was prevented from coagulation with EDTA and the samples were serially diluted in 0.9% saline. Each dilution was cultured providing a detectable threshold of 10^3^ CFU/mL.

Bacteremia synergy Assay 2:
*Group 1*: PBS sc 1 h after infection.
*Group 2*: Colistin 1 mg/kg sc at 2 and 13 h after infection.
*Group 3*: A3-APO 1 mg/kg im 1 and 12 h after infection.
*Group 4*: A3-APO 1 mg/kg im 1 and 12 h after infection, colistin 1 mg/kg sc at 2 and 13 h after infection.
*Group 5*: Colistin 10 mg/kg sc 4 h after infection, A3-APO 1 mg/kg im 6 h after infection.
*Group 6*: A3-APO 0.5 mg/kg im 1 and 12 h after infection.
*Group 7*: A3-APO 0.5 mg/kg ip 1 and 12 h after infection.
*Group 8*: A3-APO 0.5 mg/kg im 1 and 12 h after infection, colistin 1 mg/kg sc at 2 and 13 h after infection.

Survival was monitored at 12, 24, and 36 h and blood samples were taken 4 and 11 h after infection and worked up as in Assay 1.

Blood bacterial load reduction and survival in the various groups were compared with Chi-square and unpaired Student's *t*-testing, respectively (Microsoft Excel, Microsoft, 2007, Redmond, Washington, USA, and SlideWrite, Encinitas, California, USA).

Dose- and time-dependent efficacy of peptide Chex1-Arg20:

CD-1 mice of 8 weeks were infected ip with 6.8 × 10^8^ CFU/g of the extended spectrum β-lactamase producing *E. coli* 5770 strain (Szabo et al., 2010). ARV-1502 was administered ip at a 2.5, 5, and 10 mg/kg dose at 4, 8, and 12 h post-infection. Prior to drug administration at all timepoints and 4 and 20 h later (16 and 24 h post-infection), 10 μL blood was taken from the tail vein of 3 mice for determining blood bacterial counts.

#### Melioidosis model

Mean inhaled doses of 58 x LD_50_ (2 separate sprays of 56 and 60 LDs) of *Burkholderia pseudomallei* 1026b were administered to 6 to 8 week-old female Balb/c mice by whole-body aerosol. Aerosol was generated using a three-jet collision nebulizer. All aerosol procedures were controlled and monitored using the Automated Bioaerosol Exposure system (Hartings and Roy, 2004) operating with a whole-body rodent exposure chamber. Integrated air samples were obtained from the chamber during each exposure using an all-glass impinger. Mice were randomly placed into separate cages upon the conclusion of each aerosol. Cohort size for statistical evaluation was 10 mice. Ceftazidime was administered ip at 300 (*Group 1*) or 150 mg/kg (*Group 2*) doses beginning 24 h post-challenge four times a day and treatment continued for 21 days. Three additional groups receiving 150 mg/kg ceftazidime ip were treated simultaneously with 2.5, 5, or 10 mg/kg peptide ARV-1502 added im (*Groups 3–5*). A vehicle control group received 0.2 mL saline sc four times a day. Survival was monitored twice daily during treatment and once daily thereafter. Moribund animals were euthanized as necessary and counted as dead. In accordance with the protocol approved by the Institutional Animal Care and Use Committee of the United States Army Medical Research Institute of Infectious Diseases, the study was terminated at day 62. At the conclusion of the study all animals were humanely euthanized and target organs (spleens and lungs) were harvested for the determination of bacterial loads. The results were processed with a stratified Kaplan-Meyer analysis with a log-rank test as implemented on Prism Version 5.04 GraphPad.

## Results

### *In vitro* activity and synergy

**Figure 1:**
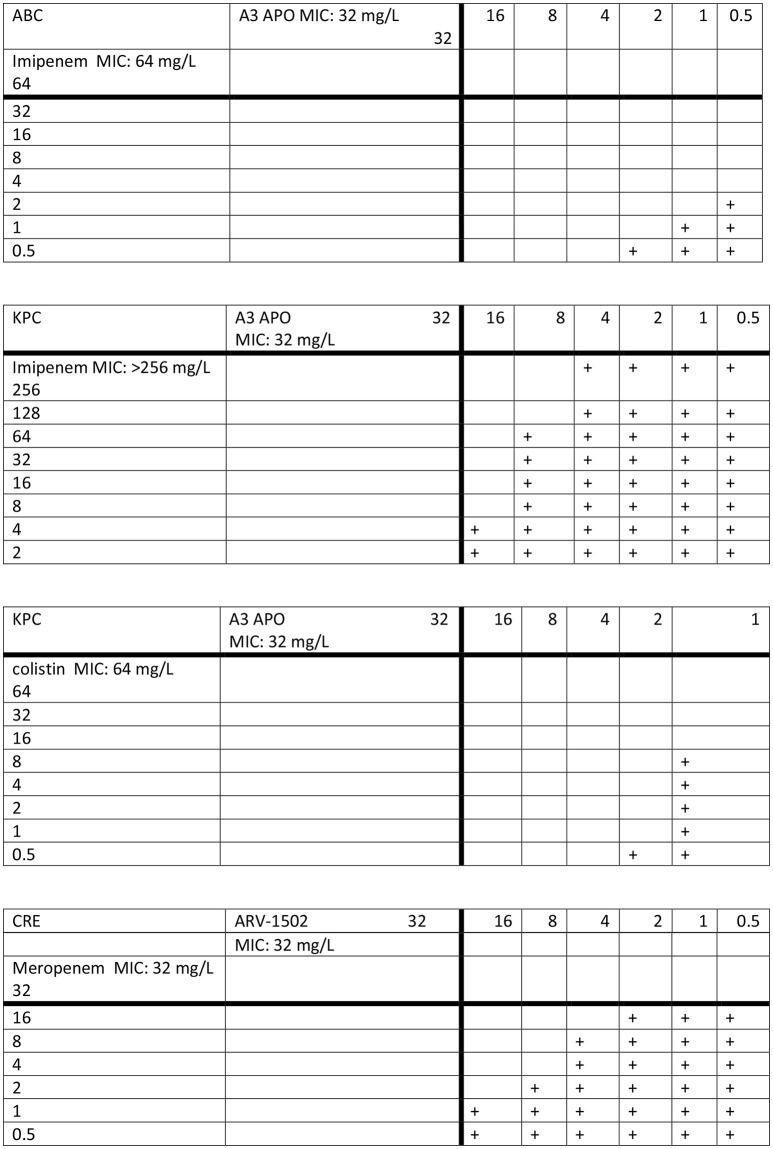
Synergy between peptide A3-APO and imipenem (top 2 panels) and colistin (third panel) against multidrug-resistant *Klebsiella pneumoniae* K97/09 (KPC, second and third panels) and *Acinetobacter baumannii* BAA-1605 (ABC, top panel) strains as well the ARV-1502 and meropenem against the carbapenem-resistant *Escherichia coli* UNT167-1 (CRE) strain (bottom panel) *in vitro*. The plus signs indicate visually visible bacterial growth in the wells. The antibiotics and the peptides were applied to bacteria in mid-log growing phase concomitantly.

MIC values of A3-APO, colistin, and imipenem against the *K. pneumoniae* strain (K97/09) were 32, 64, and > 256 mg/L, respectively. MIC values of A3-APO, colistin, and imipenem against the *A. baumannii* strain (BAA-1605) were 32, < 0.5, and 64 mg/L, respectively. Combining antimicrobials against K97/09 resulted in synergy for the A3-APO/colistin combination (ΣFIC = 0.08, Figure 1 **top panel**) and additive activity for the A3-APO/imipenem combination (ΣFIC = 0.53, Figure 1 **second panel**). Combining imipenem and A3-APO against BAA-1605 (colistin was not evaluated due to BAA-1506 being a colistin-sensitive strain) resulted in synergy for the A3-APO/imipenem combination (ΣFIC = 0.08, Figure 1 **third panel**). The negative control Allo-aca peptide or peptide Gly11, an A3-APO analog that fails to bind bacterial DnaK (Cassone et al., 2008), had no activity on either pathogen (MICs > 256 mg/L), and failed to exert any improvement in the MIC values when added together with either imipenem or colistin suggesting that the effect is specific and can be correlated with DnaK inhibition resulting in inhibition of resistance enzymes.

The MIC of both meropenem and peptide ARV-1502 against the *E. coli* UNT167-1 strain was 32 mg/L. When added together, the peptide and the carbapenem became moderately synergistic (ΣFIC = 0.38, Figure 1 **bottom panel**).

### The addition of A3-APO to colistin prolongs survival when compared to placebo

**Figure 2:**
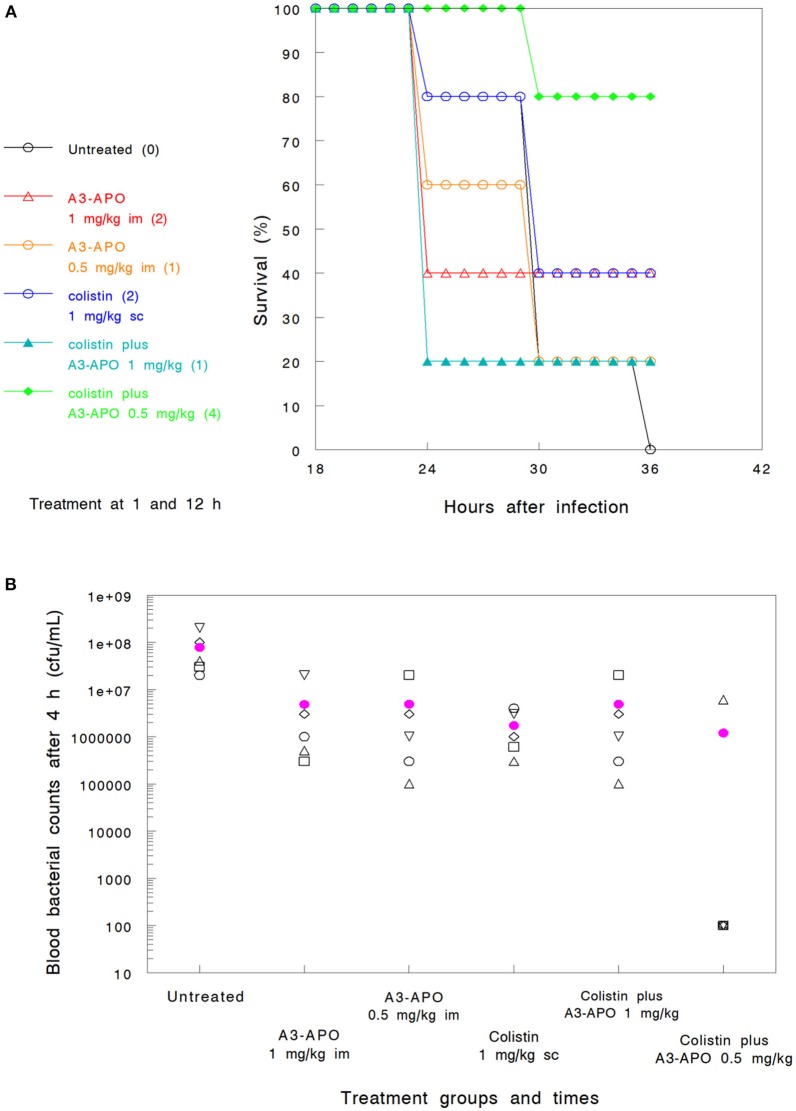
Efficacy of peptide A3-APO as a monotherapy and synergistic combinations of A3-APO results in a reduced dose of colistin and a survival advantage in a bacteremia mouse model of *Klebsiella pneumoniae* (survival, **A** and blood CFU, **B**). Treatment was administered at 1 and 12 h after infection as described in Materials and Methods. Colistin at a 1 mg/kg dose was administered subcutaneously (sc). Peptide A3-APO was added intramuscularly (im) at doses of 1 or 0.5 mg/kg. Survival was monitored after 12, 24, and 36 h of infection. The numbers in parenthesis in panel A indicate the number of surviving animals at 36 h (out of 5). The open symbols in **(B)** represent CFU/mL counts from the blood of individual mice collected 6 h after infection, the filled magenta circle is a mean of the individual mouse data. The detection limit of our assay is 10^3^ CFU/mL, all results under this value are displayed as 100.

We previously established the single agent therapeutic dose of A3-APO in a murine bacteremia infection model as 5 mg/kg im (Ostorhazi et al., 2011a). Also established was the activity, as monotherapy, of A3-APO, demonstrating a dose-dependent survival benefit (Szabo et al., 2010). In the current study, one of the experimental questions was whether lower doses than 5 mg/kg would be efficacious when used in conjunction with either colistin or imipenem in a *K. pneumoniae* bacteremia infection model. When given as monotherapy, either 0.5 mg/kg or 1.0 mg/kg im (*Groups 6 and 3 in Assay 2*) resulted in a survival advantage of 20–40%; also identified was an improvement in blood bacterial count reduction compared to untreated animals (Figures 2A,B). When administered ip, a dose of 0.5 mg/kg was even less efficacious than the same dose administered im (*Group 7 in Assay 2*, data not shown).

**Figure 3:**
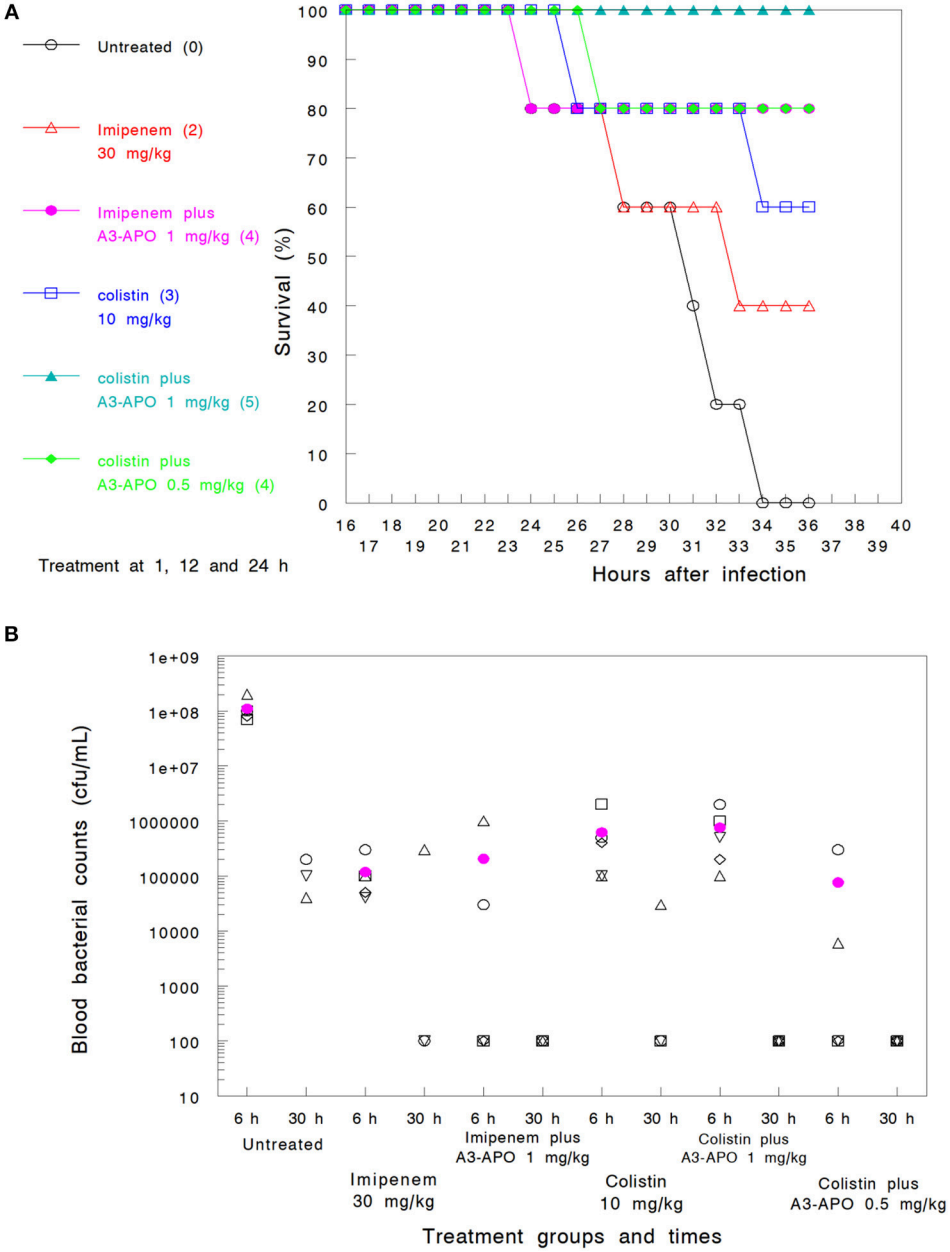
Combinations of peptide A3-APO and colistin or imipenem in a bacteremia mouse model with *Klebsiella pneumoniae* infection with survival **(A)** and blood bacterial counts **(B)** outcomes. Treatment was administered at 1, 12, and 24 h after infection as described in Materials and Methods. Imipenem (30 mg/kg) and colistin (10 mg/kg) were administered subcutaneously. Peptide A3-APO was added intramuscularly at doses 1 or 0.5 mg/kg. Survival was monitored continuously after infection. The **(A)** figures in parenthesis represent the number of surviving animals after 36 h (out of 5). The open symbols in **(B)** represent CFU/mL counts from the blood of individual mice, and the filled magenta circle is a mean of the individual mouse data. The assay detection limit is 10^3^ CFU/mL; all results under this value are displayed as 100.

*Assay 1*—*imipenem and colistin*. Imipenem administered 3 times sc at 30 mg/kg (*Group 2*) demonstrated a 40% survival improvement at 36 h over untreated controls (*Group 1*) (Figure 3A) or the 30-h blood bacterial counts (Figure 3B). Colistin administered at 10 mg/kg sc (*Group 4*) was more effective (60% survival) but had a lower blood CFU reduction (Figures 3A,B). When used in combination with 1 mg/kg A3-APO administered im 1 h prior to antimicrobial administration, a significant improvement in the 36-h survival rate was noted (80% with imipenem, *Group 3*, and 100% with colistin, *Group 5*) together with sterilization of the blood of the surviving animals at 30 h after infection (Figures 3A,B). A3-APO improved the survival rate even at the lowest dose evaluated (0.5 mg/kg im) when administered with 10 mg/kg colistin (*Group 6*, 80% at 36 h, Figure 3A). When comparing either antimicrobial with A3-APO to monotherapy arms alone, there was a significant improvement in survival (hazard ratio = 0.70, 95% CI = ±0.45; *p* < 0.005). The combination therapy was not associated with any organ toxicity. After necropsy, the weights of the heart, kidney, spleen, and liver exhibited no deviation from those of untreated control animals, or among treated groups (data not shown).

*Assay 2—reduced colistin dosing*. A 10-fold reduction in the colistin dose (*Group 2*) and lowering the frequency from thrice to twice resulted in poor comparative survival at 36 h (40%) and even less impact at reducing the bacterial burden early at the assay course (<1 log_10_ unit after 6 h, Figures 2A,B). At the lower colistin dose, A3-APO did not provide any therapeutic advantage when administered at 1 mg/kg (*Group 4*, Figure 2A). Nevertheless, a combination of a subtherapeutic dose of colistin and a low dose of 0.5 mg/kg A3-APO (*Group 8*) did improve survival (80% survival, Figure 2A). The lower dose of A3-APO (0.5 mg/kg) had a reproducibly greater effect on survival than the higher dose of A3-APO at 1.0 mg/kg. When 1 mg/kg colistin was combined with 0.5 mg/kg A3-APO under the conditions of *Assay 1* (three antibiotic doses, *Group 8*), 100% survival was observed with bacterial counts of each mice below the 10^3^ CFU/mL detection limit throughout the course of the experiment (data not shown).

### Peptide ARV-1502 exhibits extended post-antibiotic effects *in vivo*

To establish whether monotherapy with ARV-1502 protects mice in a dose-dependent manner similar to what was observed with the dimeric PrAMP, A3-APO, mice were infected with an extended spectrum β-lactamase expressing *E. coli* strain ip and treated with 2.5, 5, and 10 mg/kg of Chex1-Arg20, aka, ARV-1502 administered ip. During the 12-h treatment period a dose of 2.5 mg/kg reduced the blood bacterial load by 1.5–2 log_10_ units; by 24 h the improvement was not statistically significant (Table 1). At 5 mg/kg ARV-1502 treatment demonstrated a > 2 log10 CFU/mL reduction after inoculation and complete sterilization of the blood at 24 h. At the highest dose of 10 mg/kg, the blood was sterilized by 4 h after the last peptide treatment (Table 1). In conclusion, the monomeric PrAMP, Chex1-Arg20 (ARV-1502), has been demonstrated to be more potent than the dimeric form, requiring a lower dose than the dimeric form to achieve long-term therapeutic success (cf Holfeld et al., 2018, *in vitro*; Szabo et al., 2010, *in vivo*).

**Table 1:** Efficacy of peptide Chex1-Arg20 (ARV-1502) treatment in mice challenged intraperitoneally (ip) with *Escherichia coli* 5770 as represented by reduction of blood bacterial counts.

**Treatment**	**Bacterial counts in blood (CFU/mL) after inoculation/challenge**				
	**4 h**	**8 h**	**12 h**	**16 h**	**24 h**
Untreated	3.68 × 10^7^	6.55 × 10^7^	1 × 10^8^	2.4 × 10^7^	<3 × 10^5^
2.5 mg/kg	3.68 × 10^7^	4.2 × 10^6^ (0/3)	<1.7 × 10^6^ (1/3)	<1 × 10^6^ (1/3)	<4.8 × 10^5^ (1/3)
5 mg/kg	3.68 × 10^7^	4.3 × 10^5^ (0/3)	1.1 × 10^6^ (0/3)	<1.1 × 10^5^ (2/3)	<1 × 10^3^ (3/3)
10 mg/kg	3.68 × 10^7^	2.4 × 10^5^ (0/3)	2.9 × 10^5^ (0/3)	<1 × 10^3^ (3/3)	<1 × 10^3^ (3/3)

*The peptide was administered ip 4, 8, and 12 h after challenge (yellow box). Blood was taken immediately before antimicrobial treatments. Bacterial counts were determined from 3 mice in each group. The numbers in parentheses represent the number of animals with bacterial counts below the detection limit of 1,000 CFU/mL*.

### Long-term synergy between ARV-1502 and ceftazidime in a melioidosis model

Melioidosis is a Centers for Disease Control and Prevention (CDC) Category B bioterrorism disease and requires prolonged treatment with a high failure rate. In the murine model utilized here untreated mice died after 4 days; and Chex1-Arg20 (ARV-1502) monotherapy failed to rescue any mouse (Table 2). The 300 mg/kg therapeutic dose (allometrically scaled from 30 mg/kg human dose) of ceftazidime monotherapy resulted in a 70% survival rate at the 62-day endpoint. When the dose of ceftazidime was reduced to 150 mg/kg, survival declined to 40%. We wanted to see whether co-administration of the Chex1-Arg20 monomer can improve the treatment success at the suboptimal, reduced ceftazidime dose treatment. Co-administration of the lower ceftazidime dose of 150 mg/kg with 2.5 mg/kg ARV-1502 increased the survival rate to 60% (Table 2). Higher combination peptide doses (5 or 10 mg/kg) did not provide any survival benefit over 150 mg ceftazidime monotherapy.

**Table 2:** Treatment success in mice infected with *Burkholderia pseudomallei* 1026b.

**Antibiotic**	**Dose (mg/kg)—administration route**	**Number of deaths**	**Median survival (days post-challenge)**	***B. pseudomallei* load in survivals (CFU/spleen)—(total number of samples analyzed/samples with CFU > 10^8^)**
Untreated	Saline - sc	10	4	No survival
Ceftazidime	300 - ip	3	Undefined (>50% survival)	2.4 × 10^5^−(3/1)
Ceftazidime	150 - ip	6	56.5	2.6 × 10^5^−(4/1)
ARV-1502	5 - im	10	4	No survival
Ceftazidime + ARV-1502	150 ip + 2.5 im	4	Undefined (>50% survival)	3.1 × 10^6^−(6/1)
Ceftazidime + ARV-1502	150 ip + 5 im	6	56.5	8.6 x 10^6^−(4/0)
Ceftazidime + ARV-1502	150 ip + 10 im	7	50	7.6 x 10^6^−(3/0)

*The antibiotics were administered for 21 days, every 6 h, beginning 24 h post-challenge. After completion of the assay at day 62, surviving animals were euthanized and their spleens removed for bacterial count determination. The values in the last column are the mean of samples with < 10^8^ CFU/spleen*.

When comparing the spleen bacterial counts of surviving animals in the three groups that received ARV-1502 combinations, only the 2.5 mg/kg group had a mouse with > 10^8^ CFU raising the mean bacterial load in this (in survival terms) successful group above those in the two other (survival terms) unsuccessful treatment groups. However, removing this single outlier from the analysis (six total samples in the group) the 2.5 mg/kg peptide combination group performed best in terms of spleen bacterial load (Table 2). The spleen bacterial counts in any of peptide combination groups were not noticeably lower than those in the 150 mg/kg ceftazidime monotherapy group. Thus, once again the mortality benefit was not necessarily accompanied by a bacterial load reduction in surviving animals.

## Discussion

### *In vitro* synergy of A3-APO with legacy antimicrobials is predictive of *in vivo* activity, but not *in vivo* bacterial load reduction

The *in vitro* combination of imipenem and A3-APO against *K. pneumoniae* (K97/09) resulted in a ΣFIC of 0.53, reflecting additive effect (defined as a ΣFIC between 0.5 and 1.0), but not synergy. While survival seemed improved (40% for imipenem alone and 80% with the addition of A3-APO), the difference in survival did not reach statistical significance (*p* = 0.53). The *in vitro* combination of colistin and A3-APO against the same strain (K97/09) resulted in a ΣFIC of 0.08, reflecting synergy (defined as a ΣFIC > 0.5 ≤ 1.0). Survival was also improved with an increase from 60% at 36 h for those mice that only received colistin to 100% for mice that received a combination of colistin and 1 mg/kg A3-APO (*p* < 0.05). In either case, additive or synergistic effects, there was no significant reduction in blood bacterial load (*p* = 0.68 and 0.78, respectively). The same results were echoed in the melioidosis model: at 150 mg/kg ceftazidime and 2.5 mg/kg ARV-1502 treatment, survival improved from 40 to 60% but the spleen bacterial counts of the surviving animals were not lower that with 150 mg/kg ceftazidime treatment alone.

### Biochemical basis of synergy *in vitro*

Based on results with a peptide analog that fails to bind bacterial DnaK (Gly11) and an unrelated control peptide (Allo-aca), the synergistic effect of A3-APO is sequence-specific. The inhibitory effect, therefore, is likely reflective of improper protein folding, including housekeeping proteins and possibly those proteins required for antimicrobial resistance. The MIC of both PrAMPs was also reduced when used in combination with antibiotics. The rate-limiting step in our PrAMP activity, including the ARV-1502 monomer (as well as its oligomers), is penetration across bacterial membranes (Li et al., 2016). Colistin and carbapenems kill bacteria by interfering with bacterial membrane assembly (Bialvaei and Samadi Kafil, 2015; Pitout et al., 2015) and therefore, even small reductions in bacterial membrane integrity will help PrAMP actions. The PrAMP monomer, ARV-1502, may have advantage over the prodrug dimer in a combination therapy as it permeates bacterial membranes less than the dimeric form, but binds DnaK more avidly (Cassone et al., 2008; Li et al., 2016).

### Treatment failure rescue

Two groups of mice were tested to determine if single dose treatment with colistin monotherapy can be enhanced with the later addition of 1 mg/kg single dose A3-APO. In *Assay 1* (*Group 9*), when colistin was administered soon after infection (2 h), all 5 mice survived, and blood was subsequently sterilized. In *Assay 2* (*Group 5*), when 10 mg/kg colistin was administered after 4 h post-bacterial exposure, only 1 mouse survived and the blood bacterial counts at 6 h post-infection were not lower than those of untreated controls (*Group 1*). In this model, when bacteremia is established and colistin fails to impact survival, A3-APO administration does not improve outcome. This finding contrasts with those presented in Figure 3, related to PrAMP administration soon after bacterial exposure. The advantage of “priming” HDP prior to antimicrobial administration rather than after antibiotic administration was documented recently in a murine *A. baumannii* bacteremia and transdermal peptide dosing model (Ostorhazi et al., 2017).

### Alternative modes of action *in vivo*

Unexpectedly, while a mortality benefit was observed with the addition of A3-APO to colistin or ARV-1502 to ceftazidime, a reproducible parallel benefit in bacterial load reduction was not observed. Given that the mechanism of action of the APO-type HDP is non-membrane disruptive, actual bacterial killing, or even the potentiation of bacterial killing, may not be the life-preserving benefit of A3-APO or ARV-1502. The data suggest that the legacy antibiotics are responsible for direct killing of the bacteria.

In prior studies, A3-APO was noted to be immunostimulatory and anti-inflammatory *in vitro* (Ostorhazi et al., 2011b; Otvos et al., 2014). Inhibition of DnaK disrupts the heat shock response of bacteria and has repeatedly been shown to improve the outcome of small molecule chemotherapy against a series of pathogens and across several antibiotic classes (Yamaguchi et al., 2003; Evans et al., 2010). It may be that at the time points assessed, DnaK inhibitory actions predominate, resulting in a mortality benefit without a concomitant reduction in bacterial burden. The increased activity of early PrAMP intervention compared to later treatment post-bacterial challenge supports this hypothesis.

Surprising was the observation that the 0.5 mg/kg A3-APO dose improved survival more so than the 1.0 mg dose, in combination with a subtherapeutic dose of colistin. Furthermore, the 2.5 mg/kg dose of ARV-1502 improved survival over the 5 and 10 mg/kg doses in combination with the subtherapeutic dose of ceftazidime (150 mg/kg). This may reflect an element of hormesis since historically, concomitant use of toxic antimicrobials in some infection models have resulted in worse outcomes (Zou et al., 2013). The enhanced activity at the lower dose may reflect the serendipitous identification of a dose within a hormetic range. Significantly, decreased doses of drugs were shown to protect mammals with modes of action different from those brought upon applying increased doses (Calabrese, 2008a). At 5 mg/kg (in mice) the APO peptides may directly influence bacterial proliferation and at lower doses their mechanism of action may shift, possibly to an immunostimulatory function (Ostorhazi et al., 2013; Otvos, 2016). When combined with small molecule antibiotics, bacterial killing may be a consequence of the antibiotic with immune augmentation from the PrAMP. In the extended course of melioidosis, ARV-1502-mediated inactivation of released bacterial toxins (through DnaK inhibition) can also improve survival similarly to the toxin inhibitory effect of A3-APO observable in a long-term murine model of anthrax (Otvos et al., 2014). The major bacterial target might be the *Burkholderia* lethal factor 1 toxin that inactivates the translation factor eIF4A helicase (Cruz-Migoni et al., 2011).

At a 1 mg/kg dose, the full-body concentration of our peptides cannot exceed 1 μg/g, a value clearly lower than the MIC even against highly sensitive strains and without calculating in the poor pharmacokinetics of peptide drugs. At this low dose, the peptides should not comply with the suggested 1.3 x MIC in the circulation (Bush et al., 2004). Likewise, the 0.5–1 μM maximum concentration is only slightly above the measured affinity figures of A3-APO/DnaK or Chex1-Arg20/DnaK complexes at the molecular level (Zahn et al., 2013). It needs to be added, however, that the X-ray measure detected peptide binding only to the substrate binding pocket, an association that seems to be weaker than interaction with the C-terminal multihelical lid of DnaK in sensitive bacteria (Kragol et al., 2001). Thus, the two expected modes of actions, and those that have been proven to dominate at higher doses (5–20 mg/kg) in various animal models (Ostorhazi et al., 2010; Szabo et al., 2010; Otvos et al., 2014) cannot apply here. We are left with a shift to immunostimulatory effects, a hypothesis that requires exploration; experiments are currently underway in our laboratories to further such understanding. In support, immune-system-related hormetic-like biphasic dose-response relationships are common but so far have been only little appreciated (Calabrese, 2008b). Our knowledge of immunological responses influencing complex regulatory networks and affecting biological switching mechanisms that result in the hormetic responses are rapidly expanding with insights into sub-MIC antibiotics and heat shock response modifiers involved (Dattilo et al., 2015; Mathieu et al., 2016).

### Risk of resistance induction

Historically, AMP/HDP were considered of low risk to induce resistance in microbiology terms (Zasloff, 2002). However, if the dose is reduced and not all bacteria are killed such as in our combination therapy, the residual bacterial burden can lead to genetic mutations leading to resistance induction. Having said this, at the applied doses the modes of action of A3-APO or Chex1-Arg20 are expectedly different than direct killing of bacteria. The question remains whether other resistance mechanisms can come into picture. DnaK being a housekeeping protein it is unlikely to go through genetic mutations. Indeed, DnaK mutants of *Staphylococcus aureus* are increasingly susceptible to oxidative and cell-wall-active antibiotic stress conditions (Singh et al., 2007). Perhaps more alarming, *in vitro*, resistance to the first AMP in clinical trials, pexiganan, makes *S. aureus* resistant to a defensin that serves a key component of the innate immune response to infection (Habets and Brockhurst, 2012). In general, undermining of the innate immune system projects potential drawbacks of clinical AMP/HDP therapies (Otvos and Ostorhazi, 2015).

### Potential pharmaceutical advantages

No Food and Drug Administration-approved antimicrobial carries a resistance indication for current Gram-negative threats including carbapenem-resistant Enterobacteriaceae, extended-spectrum β-lactamases, or colistin-resistant isolates. Most importantly, no novel antibiotic has a labeled clinical superiority over legacy antimicrobials. Use of modified PrAMPs, either Chex1-Arg 20 (ARV-1502) or A3-APO as adjuncts to care standards provide two salient advantages over historic drug development programs. The therapeutic window of legacy antibiotics can be expanded, making antibiotics with well-characterized benefit/risk profiles more clinically useful despite growing antibiotic resistance and such peptides can augment the efficacy of co-administered antibiotics by inhibiting a series of bacterial chaperone protein functions.

## Author contributions

LO, EO, SZ, and CK assay design, data analysis, and manuscript preparation. EO, DS, LM, SH, PD, and SI *in vitro* and animal studies.

### Conflict of interest statement

CK is the Chief Executive Officer of Arrevus, Inc., a biotechnology company focusing on the clinical development of the APO-type proline-arginine-rich hose defense peptides. LO is an advisor to Arrevus and is the inventor of an issued patent on the Chex1-Arg20 peptide that is licensed by Arrevus. CK and LO are inventors on a new provisional patent application describing the use of A3-APO in combination with colistin and imipenem. The remaining authors declare that the research was conducted in the absence of any commercial or financial relationships that could be construed as a potential conflict of interest.
