# Supplementary material for: Synergy Between Proline-Rich Antimicrobial Peptides and Small Molecule Antibiotics Against Selected Gram-Negative Pathogens in vitro and in vivo
Source: Front Chem. 2018 Aug 14;6:309. doi: 10.3389/fchem.2018.00309 (PMC6102830; doi:10.3389/fchem.2018.00309)
Supplement: Supplementary file 1 [file Data_Sheet_1.pdf]

Supplementary Material

Treatment schedule in bacteremia Assays 1 (top) and 2 (bottom)

Group	Treatment	Dosing (hour)						
		1	2	5	13	14	25	26
1	PBS (sc)	■						
2	Imipenem (30 mg/kg sc)		■			■		■
3	Imipenem (30 mg/kg sc)		■			■		■
	A3-APO (1.0 mg/kg im)	■			■		■	
4	Colistin (10 mg/kg sc)		■			■		■
5	Colistin (10 mg/kg sc)		■			■		■
	A3-APO (1.0 mg/kg im)	■			■		■	
6	Colistin (10 mg/kg sc)		■			■		■
	A3-APO (0.5 mg/kg im)	■			■		■	
7	Colistin (1.0 mg/kg sc)		■			■		■
	A3-APO (1.0 mg/kg im)	■			■		■	
8	Colistin (1.0 mg/kg sc)		■			■		■
	A3-APO (0.5 mg/kg im)	■			■		■	
9	Colistin (10 mg/kg sc)		■					
	A3-APO (1.0 mg/kg im)			■				

Group	Treatment	Dosing (hour)					
		1	2	4	6	12	13
1	PBS (sc)	■					
2	Colistin (1.0 mg/kg sc)		■				■
3							
	A3-APO (1.0 mg/kg im)	■				■	
4	Colistin (10 mg/kg sc)		■				■
	A3-APO (1.0 mg/kg im)	■				■	
5	Colistin (10 mg/kg sc)			■			
	A3-APO (0.5 mg/kg im)				■		
6							
	A3-APO (0.5 mg/kg im)	■				■	
7							
	A3-APO (0.5 mg/kg ip)	■				■	
8	Colistin (1.0 mg/kg sc)		■				■
	A3-APO (0.5 mg/kg im)	■				■	
